# Integrated Transcriptomic and Biochemical Profiling Reveals the Regulatory Mechanism of Light Intensity-Induced Anthocyanin Biosynthesis in a Purple-Leaf Tea Cultivar

**DOI:** 10.3390/plants15182805

**Published:** 2026-09-13

**Authors:** Wei Li, Xiaoqin Tan, Qian Tang

**Affiliations:** 1 Faculty of Agriculture, Forestry and Food Engineering, Yibin University, Yibin 644005, China; 2 College of Horticulture, Sichuan Agricultural University, Chengdu 611130, China; 3 Tea Resources Utilization and Quality Testing Key Laboratory of Sichuan Province, Chengdu 611130, China

**Keywords:** light intensity, catechin, anthocyanin biosynthesis, transcription factors, purple-leaf tea (*Camellia sinensis* (L.) O. Kuntze)

## Abstract

Anthocyanins are water-soluble flavonoid pigments that contribute to leaf coloration, tea quality, and have potential health-promoting properties. Purple-leaf tea plants have attracted significant interest due to their high anthocyanin levels; however, cultivar-specific evidence linking light intensity with pigment accumulation, enzyme activity, and transcriptional regulation in purple-leaf tea remains limited. In this study, the purple-leaf tea cultivar ‘Ziyan’ was exposed to three light intensity treatments: low (LL, 100 μmol·m^−2^·s^−1^), medium (ML, 200 μmol·m^−2^·s^−1^), and high (HL, 400 μmol·m^−2^·s^−1^). Increasing light intensity resulted in deeper purple coloration and significantly higher anthocyanin accumulation. The contents of delphinidin, cyanidin, pelargonidin, and total anthocyanins under HL increased by 124.2%, 63.4%, 57.2%, and 105.9%, respectively, compared to the control. Activities of major biosynthetic enzymes (CHS, CHI, F3H, F3′H, F3′5′H, DFR, ANS) were enhanced under ML and HL, whereas ANR activity decreased. Transcriptome sequencing identified 1349 differentially expressed genes (DEGs) (404 up-regulated, 945 down-regulated) in the LL vs. HL comparison. Notably, TFBS motif enrichment analysis revealed that up-regulated DEGs were dominated by a single cohesive SPL/SBP regulatory module, while down-regulated DEGs partitioned into independent NAC, HSF, and EIL modules, suggesting a multi-layered transcriptional regulatory network governing light-responsive anthocyanin biosynthesis. Several transcription factors, including MYB44, MYB75, bHLH162, and WRKY40, were also identified as candidate regulators. These results indicate that increasing light intensity is associated with enhanced anthocyanin accumulation, accompanied by coordinated changes in phenotype, flavonoid metabolism, enzyme activities, and gene expression, providing evidence for a possible regulatory mechanism of light-responsive pigmentation in purple-leaf tea and a basis for cultivation optimization and molecular breeding.

## 1. Introduction

Tea (*Camellia sinensis*) is one of the most widely consumed non-alcoholic beverages in the world and an economically important perennial crop. The chemical composition of tea leaves is a key determinant of the quality and flavor of final products. Purple-leaf tea germplasm has attracted increasing interest because of its distinctive appearance, elevated anthocyanin content, and potential value for specialty tea development, functional ingredient utilization, and germplasm innovation. Anthocyanins can accumulate to levels substantially higher than those in conventional green-leaf tea cultivars, making purple tea important for the study of both tea quality formation and flavonoid regulation [1].

Light is one of the most important environmental signals controlling plant pigmentation, and light intensity is the most influential attribute for anthocyanin biosynthesis [2]. In tea plants, the light intensity affects not only leaf coloration but also the accumulation of flavonoids, catechin, amino acids, chlorophyll, and aroma-related metabolites, making light management both a fundamental biological mechanism and a practical agronomic tool for quality regulation [3,4,5,6]. Shading generally attenuates flavonoid biosynthesis and reduces anthocyanin accumulation in purple-leaf cultivars, whereas enhanced light stimulates anthocyanin accumulation in purple-bud tea plants [7] and in other species such as rough bluegrass [8], mangosteen [9] and *Aglaonema commutatum* [10,11]. These observations collectively identify light intensity as a key environmental lever on flavonoid metabolism, although the quantitative response of a defined cultivar to a controlled light intensity gradient remains insufficiently characterized.

Anthocyanins are synthesized through a branch of the flavonoid pathway: phenylalanine is converted by the general phenylpropanoid enzymes phenylalanine ammonium lyase (PAL), cinnamate 4-hydroxylase (C4H), and 4-coumarate—CoA ligase (4CL) to p-coumaroyl-CoA, which is successively processed by chalcone synthase (CHS), chalcone isomerase (CHI), flavanone 3-hydroxylase (F3H), dihydroflavonol 4-reductase (DFR), and anthocyanidin synthase (ANS) to anthocyanidins, and finally glycosylated by UDP-dependent anthocyanidin-3-O-glucosyltransferase (3GT) [12]. Transcriptional control is exerted mainly by the MYB–bHLH–WD40 (MBW) activation complex and its regulators [13,14,15]. In tea plants, CsMYB5a and CsMYB5e, CsMYB75, CsMYB86, the negative regulators CsMYBL2a/b, and CsRAB, together with the light-responsive bZIP factor CsHY5 and several bHLH and WRKY factors, have been implicated in the regulation of anthocyanin and catechin biosynthesis [16,17,18,19,20,21]. At the post-transcriptional level, the miR156–SPL module has been shown to fine-tune anthocyanin biosynthesis in *Arabidopsis* [22] and, more recently, in tea plants [23].

Although considerable advances have been made in previous research—for example, Song et al. [24] systematically compared flavonoid metabolites and related gene expression profiles between purple and green leaves of the purple-tea cultivar ‘Zijuan’ using an integrated metabolome and transcriptome approach, and elucidated key mechanisms regulating flavonoid and anthocyanin biosynthesis—two important gaps persist that deserve further investigation. First, most previous studies of tea have focused either on shading effects, single regulators, or comparisons between purple and green germplasm, whereas the response of a defined purple-leaf cultivar to a controlled light intensity gradient has not been sufficiently characterized. Second, many reports describe either metabolite changes or gene expression responses, making it difficult to evaluate how visible coloration, anthocyanin composition, competing flavonoid branches, enzyme activities, and transcriptional regulation change in a coordinated manner under the same experimental system. These limitations weaken both the mechanistic interpretation and the practical value of existing knowledge for cultivation management.

The objective of this study was therefore to combine phenotypic, biochemical (anthocyanin and catechin quantification and enzyme activity assays), and transcriptomic (RNA-seq, qRT-PCR, and TFBS motif enrichment) analyses to elucidate how light intensity regulates anthocyanin and catechin accumulation in the purple-leaf tea cultivar ‘Ziyan’, and to identify candidate regulatory modules underlying this response. The results are expected to explain how external light cues are translated into coordinated changes in structural genes, regulatory factors, and pathway flux; to provide a reliable basis for light-management optimization in purple-tea cultivation; and to identify stable light-responsive candidate genes supporting germplasm evaluation and molecular breeding of anthocyanin-rich tea cultivars.

## 2. Results

### 2.1. Color Analysis of New Shoots

**Figure 1:**
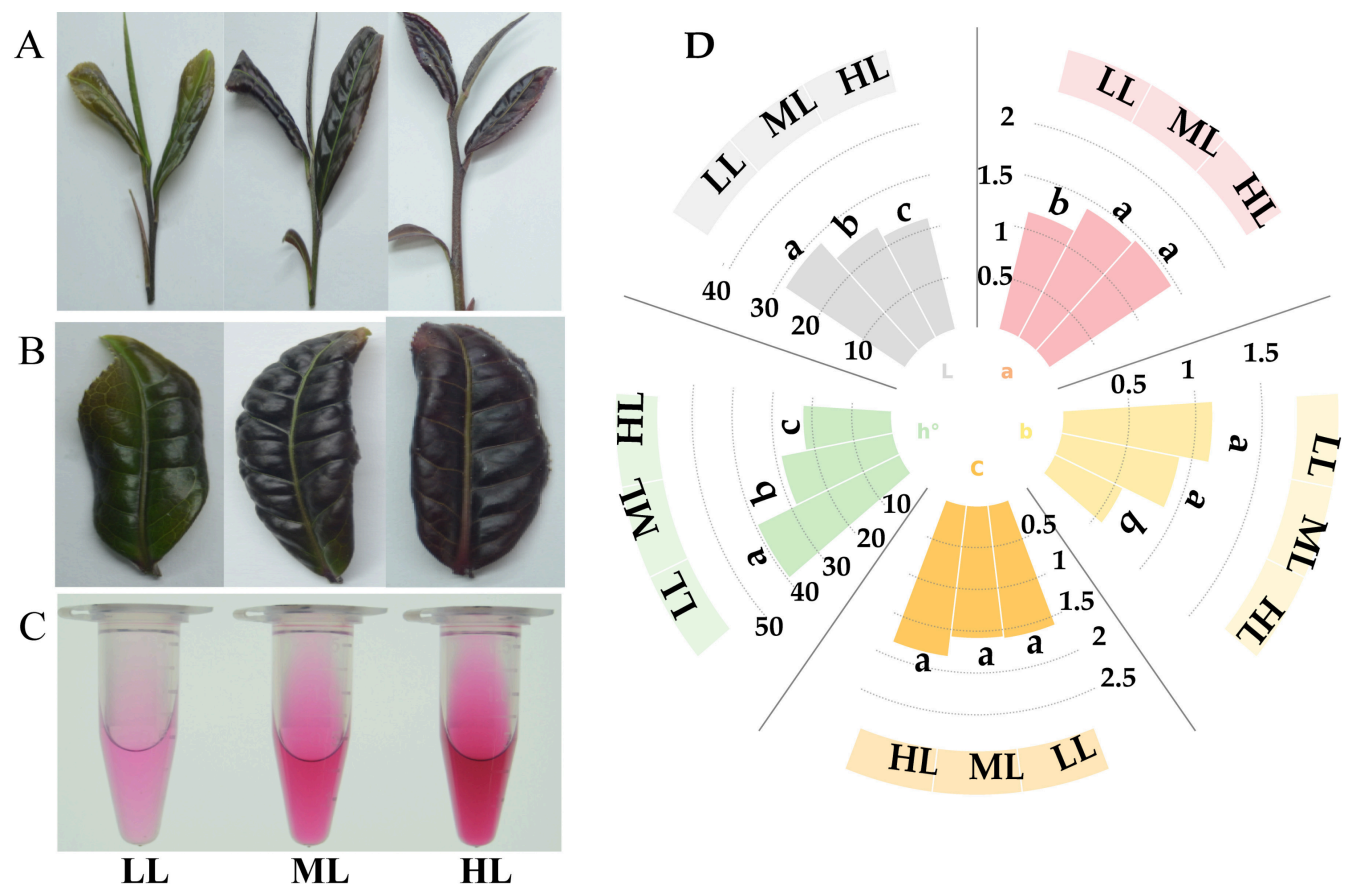
Color analysis of new shoots under different light intensity treatments. (**A**) One bud and two leaves of a new shoot; (**B**) the top second leaf of ‘Ziyan’; (**C**) methanolic anthocyanin extract from one bud and two leaves; and (**D**) color values of the top second leaves. LL, ML, and HL represent low-light-intensity, medium-light-intensity, and high-light-intensity treatments, respectively. Different lowercase letters above the bars indicate significant differences (*p* < 0.05). Color was recorded in the L a b color space, where L represents lightness, a indicates the green (−)-to-red (+) spectrum, and b represents the blue (−)-to-yellow (+) spectrum. The chroma (C) and hue angle (h°) were also calculated.

With increasing light intensity, the color of the new shoots and leaves of the ‘Ziyan’ tea cultivar darkens and becomes more purple (Figure 1A,B); correspondingly, the methanolic anthocyanin extracts prepared from one bud and two leaves deepened visibly in color (Figure 1C). The color of the second leaf from the top of one bud and two leaves was measured using a colorimeter, and the results are expressed in *L a b* and *L C h°* color space values. The *L*, *b*, and *h*° values across all light intensity treatments followed the trend LL > ML > HL, with the values for LL, ML, and HL being (L: 28.6, 24.30, 22.18; b: 1.13, 0.91, 0.57; h°: 39.54, 28.67, 22.31), respectively. Statistically significant differences (*p* < 0.05) were found between the *L* and *h*° values in the different treatments, indicating that leaf color brightness was higher under low-light-intensity conditions and diminished under high-light-intensity conditions (Figure 1). Compared to low light intensity, the *a* values in the medium- and high-light-intensity treatments increased significantly (*p* < 0.05) by 24.79% and 21.36%, respectively. No significant difference (*p* > 0.05) was observed between high and medium light intensities. Furthermore, no significant differences (*p* > 0.05) in the C values were detected between the treatments.

### 2.2. Transcriptome Sequencing and Gene Mapping

To elucidate the molecular mechanism underlying anthocyanin accumulation in the young shoots of ‘Ziyan’ in response to varying light intensity treatments, we employed high-throughput RNA-Seq. Complementary DNA (cDNA) libraries from six samples were sequenced (Table S1). A total of 125.13 Gb of clean bases was obtained using the Illumina HiSeq 4000 platform. The GC content and Q30 bases exceeded 45% and 92%, respectively. The mapping ratio of clean reads to the tea plant reference genome ranged from 87.15% to 89.24% (Table S1). The reproducibility of each treatment was good, with correlation coefficients exceeding 0.94 (Figure S1). A total of 46,585 expressed genes were mapped to the genome. In this study, we identified 12,655 novel genes, of which 9435 were functionally annotated based on comparative analysis, variable splicing prediction, and genetic structure optimization analysis. The reliability of the RNA-seq data was validated by quantitative reverse transcription polymerase chain reaction (qRT-PCR). The relative expression levels of 12 selected genes were consistent with the RNA-seq results (Figure S2).

### 2.3. Analysis of Differentially Expressed Genes (DEGs)

**Figure 2:**
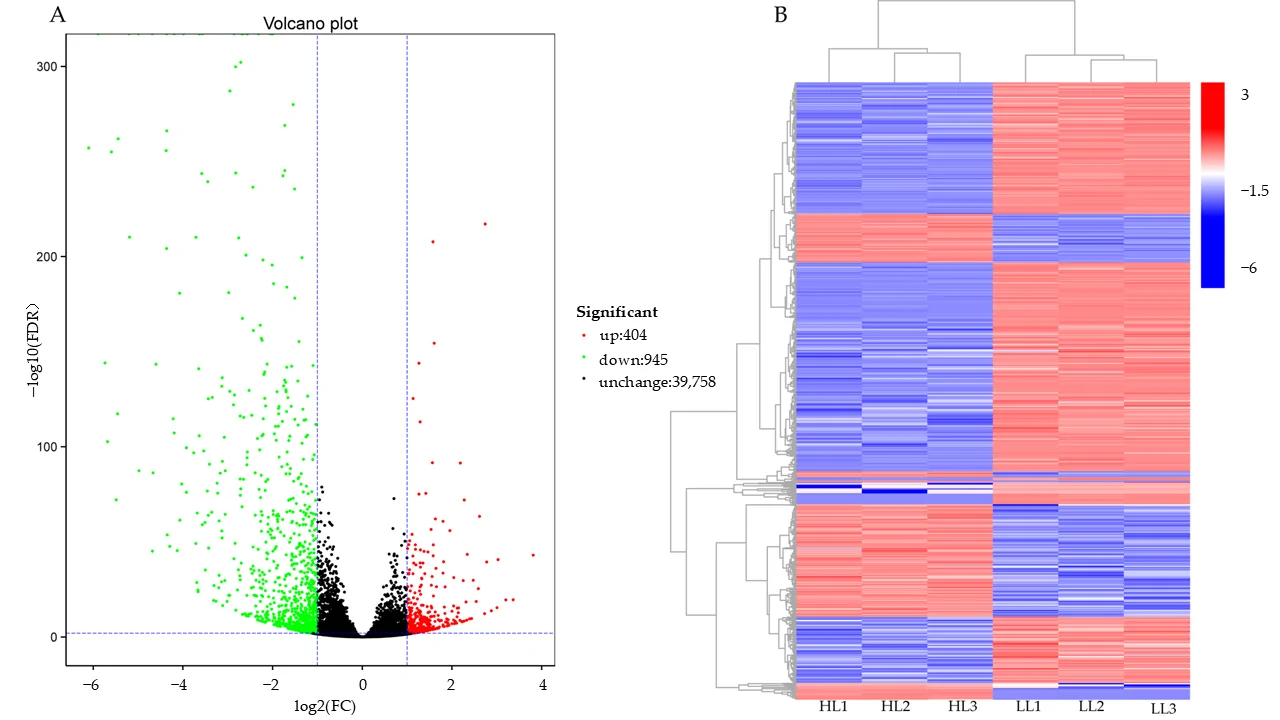
Differential gene expression analysis for the LL vs. HL comparison. (**A**) Volcano plot of differentially expressed genes; (**B**) hierarchical clustering heatmap of differentially expressed genes.

To investigate transcriptional responses to varying light intensities, differentially expressed genes (DEGs) were identified between the HL and LL treatments. A total of 1349 DEGs were detected in the LL vs. HL comparison (with LL serving as the control), comprising 404 up-regulated and 945 down-regulated genes under high-light-intensity conditions (Figure 2A). Hierarchical clustering analysis revealed high reproducibility among biological triplicates within each treatment group, while transcriptome profiles exhibited distinct separation between the two light intensity conditions (Figure 2B). These findings suggest that light intensity significantly modulates gene expression in young shoots. To further characterize their potential functions, all DEGs were functionally annotated using multiple public databases, including KEGG, COG, Nr, KOG, Pfam, Swiss-Prot, GO, and eggNOG (Table S2).

To further elucidate the functional implications of the identified DEGs, Gene Ontology (GO) and Kyoto Encyclopedia of Genes and Genomes (KEGG) enrichment analyses were conducted. The GO enrichment analysis was performed for DEGs from the LL vs. HL comparison, and the results are summarized in Figure S3. These DEGs were categorized into three main ontologies: biological process (BP), cellular component (CC), and molecular function (MF). Within the BP category, DEGs fell into 18 subcategories, clustering predominantly within “metabolic process,” “cellular process,” “single-organism process,” “biological regulation,” and “response to stimulus.” For the CC domain, 11 subcategories were identified, primarily associated with “cell part,” “cell,” “organelle,” and “membrane.” In the MF category, DEGs were distributed across 12 subcategories, with “catalytic activity,” “binding,” and “transporter activity” being the most prevalent subcategories.

Additionally, KEGG pathway enrichment analysis revealed that DEGs in the LL vs. HL group mapped onto 93 distinct pathways. Most relevant to the objective of this study, phenylpropanoid biosynthesis—the upstream pathway supplying the flavonoid/anthocyanin branch—was among the most significantly enriched pathways, together with nitrogen metabolism, plant hormone signal transduction, galactose metabolism, monoterpenoid biosynthesis, and photosynthesis antenna proteins (Figure S4). In the GO analysis, the enriched biological-process terms likewise clustered in ‘metabolic process’ and ‘response to stimulus’, pointing to a coordinated remodeling of secondary metabolism under high light intensity.

### 2.4. Transcription Factor (TF) Analysis

**Figure 3:**
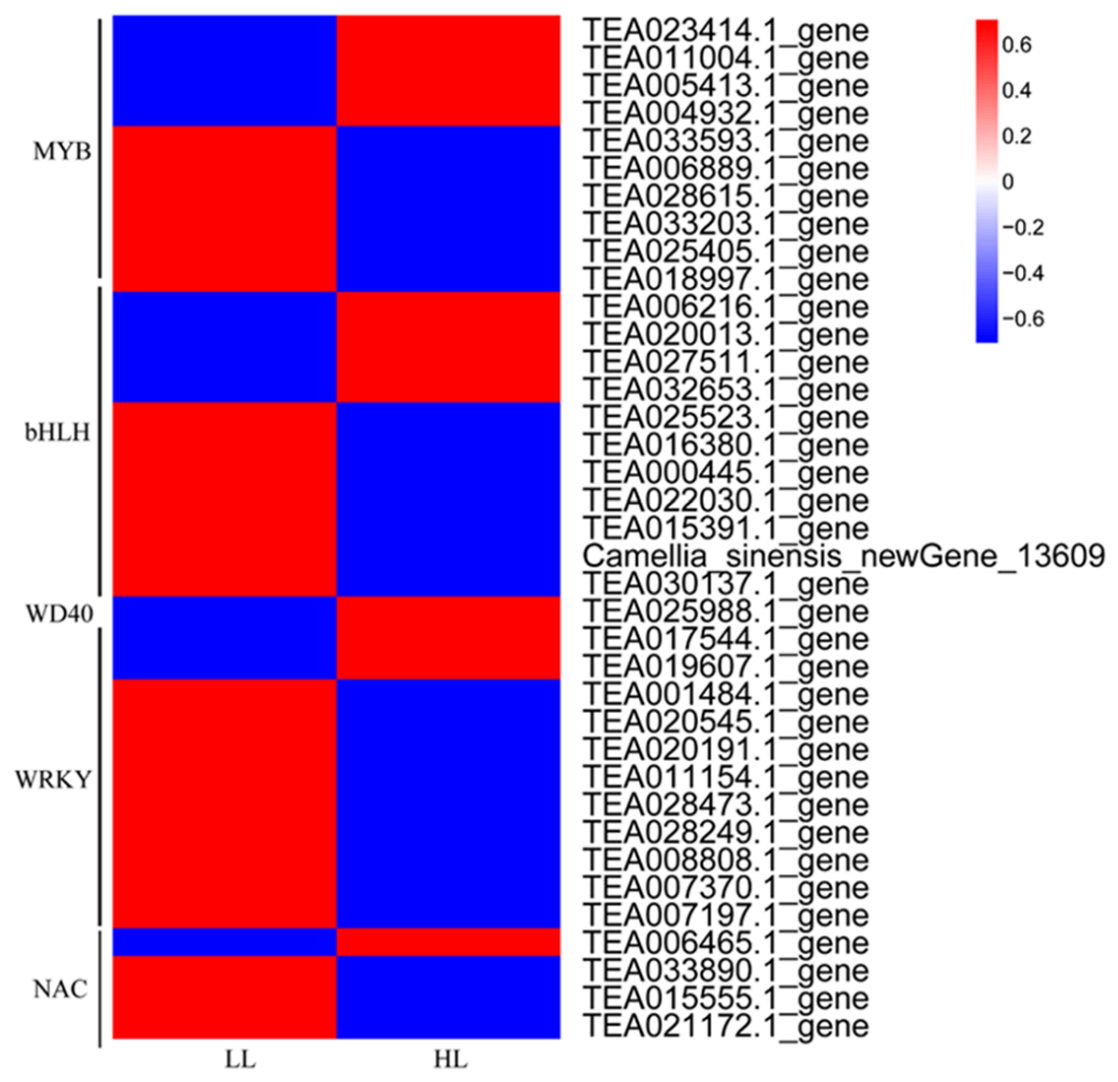
Expression patterns of transcription factors in differentially expressed genes (LL vs. HL).

It has been well established that the primary transcriptional regulators of anthocyanin biosynthesis include TFs from the MYB, bHLH, and WRKY families, together with WD40 repeat proteins. In this study, relevant genes were identified through the annotation of differentially expressed genes (Figure 3), and potential transcription factors involved in the regulation of anthocyanin transcription were further analyzed based on previous research. Specifically, these include MYB family members MYB44, MYB14, MYB61, MYB73, MYB75, MYB82, MYB48, and MYB102; bHLH family members bHLH137, bHLH162, bHLH92, bHLH51, GL3, and bHLH35; WRKY family members WRKY11, WRKY23, WRKY40, and WRKY71; and NAC family member NAC83. These findings indicate that changes in light intensity are associated with marked changes in the expression of candidate TF regulators in tea shoots.

### 2.5. Analysis of Enrichment with Binding Sites for TFs

**Figure 4:**
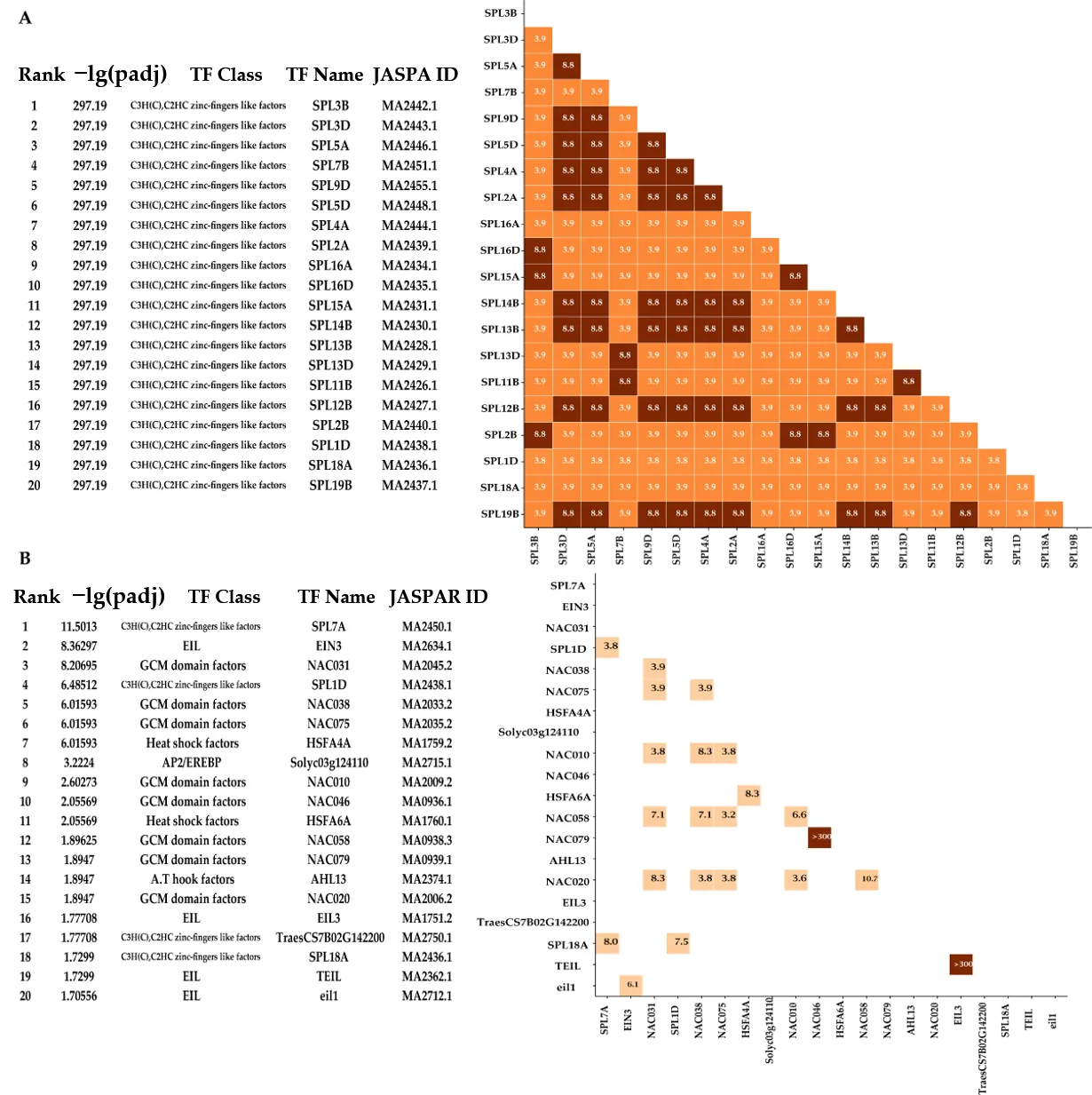
TFBS motif enrichment among the top-20 enriched motifs in the 1 kb promoters of (**A**) up-regulated and (**B**) down-regulated DEGs. The left panel lists, for each motif, its rank, enrichment significance [−log10(padj)], TF class, TF name, and JASPAR motif ID; the right panel shows pairwise motif similarity (−log10(*p*)), where empty cells indicate no significant overlap (*p* > 0.05) and the shades from light orange to dark orange denote significantly similar motifs.

TFBS motif enrichment in the 1 kb promoters of DEGs revealed a pronounced asymmetry between the up- and down-regulated regulatory programs (Figure 4). In the up-regulated set (Figure 4A, Tables S3 and S4), all 20 top-enriched motifs belonged to the SPL/SBP (SQUAMOSA promoter-binding protein) family and formed a single cohesive module, with pairwise −log10(*p*) values ranging from 3.75 to 8.76. In contrast, the down-regulated set (Figure 4B) partitioned into four distinct modules—a NAC module (strongest pair NAC058 × NAC020, −log10(*p*) = 10.73), an HSF module (HSFA4A × HSFA6A, 8.28), an EIL module (EIN3 × eil1, 6.11; EIL3 × TEIL, >300, i.e., identical target sets), and a non-overlapping SPL module (SPL7A/SPL1D/SPL18A, 7.5–7.98)—whereas AHL13 and Solyc03g124110 showed no significant pairwise overlap with any module. Collectively, these motif modules delineate a compact set of candidate TF cohorts that distinguish the up- and down-regulated transcriptional branches of the light intensity response.

### 2.6. Different Expression Profiles of Genes Associated with Anthocyanin Biosynthesis

**Figure 5:**
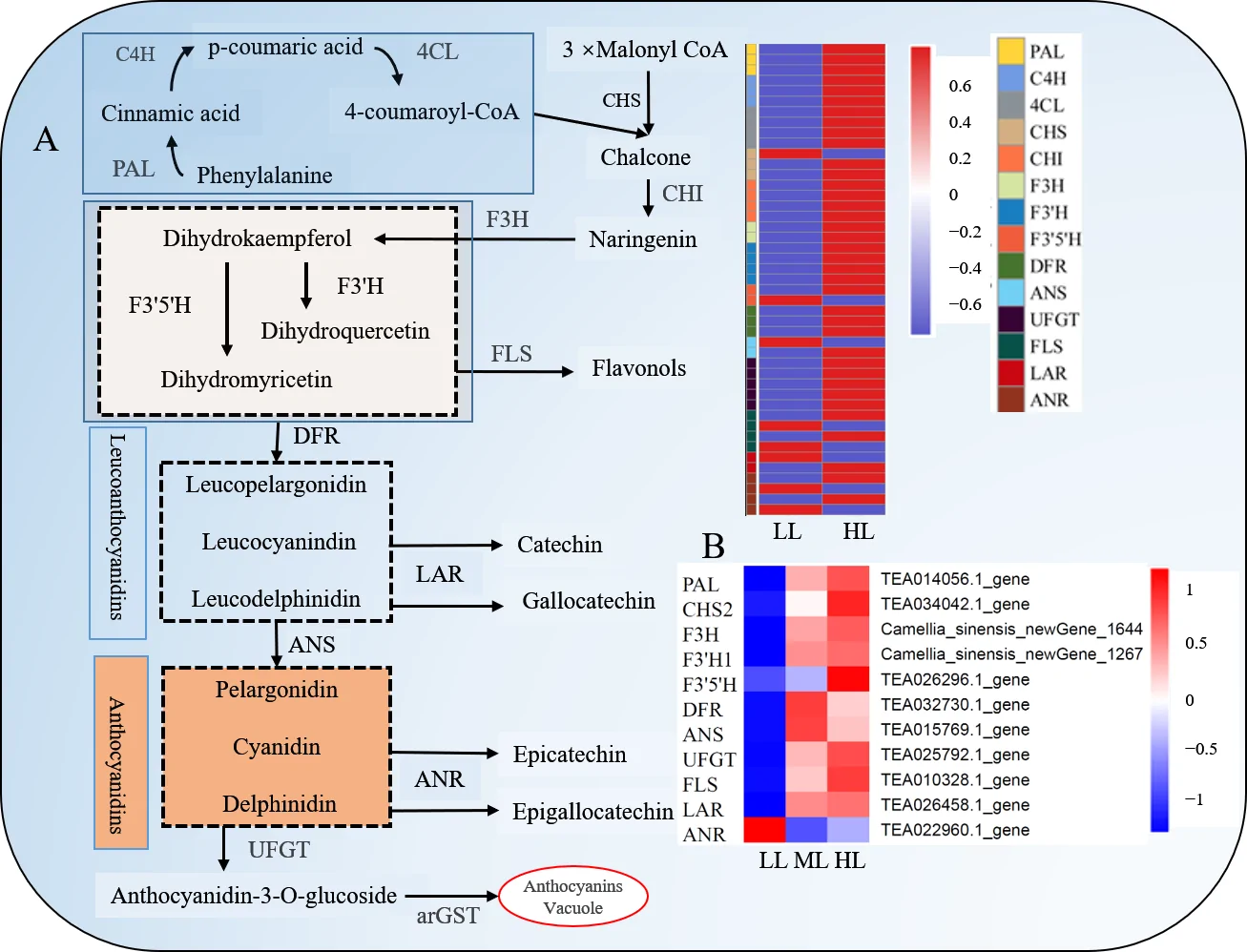
Expression patterns of structural genes in the anthocyanin synthesis pathway. (**A**) Anthocyanin biosynthetic pathway and heatmap of normalized transcriptomic data; (**B**) heatmap of normalized qRT-PCR relative expression data. The color scale from blue to red indicates relative expression levels from low to high, respectively. LL, ML, and HL represent low, medium, and high-light-intensity treatment, respectively.

**Figure 6:**
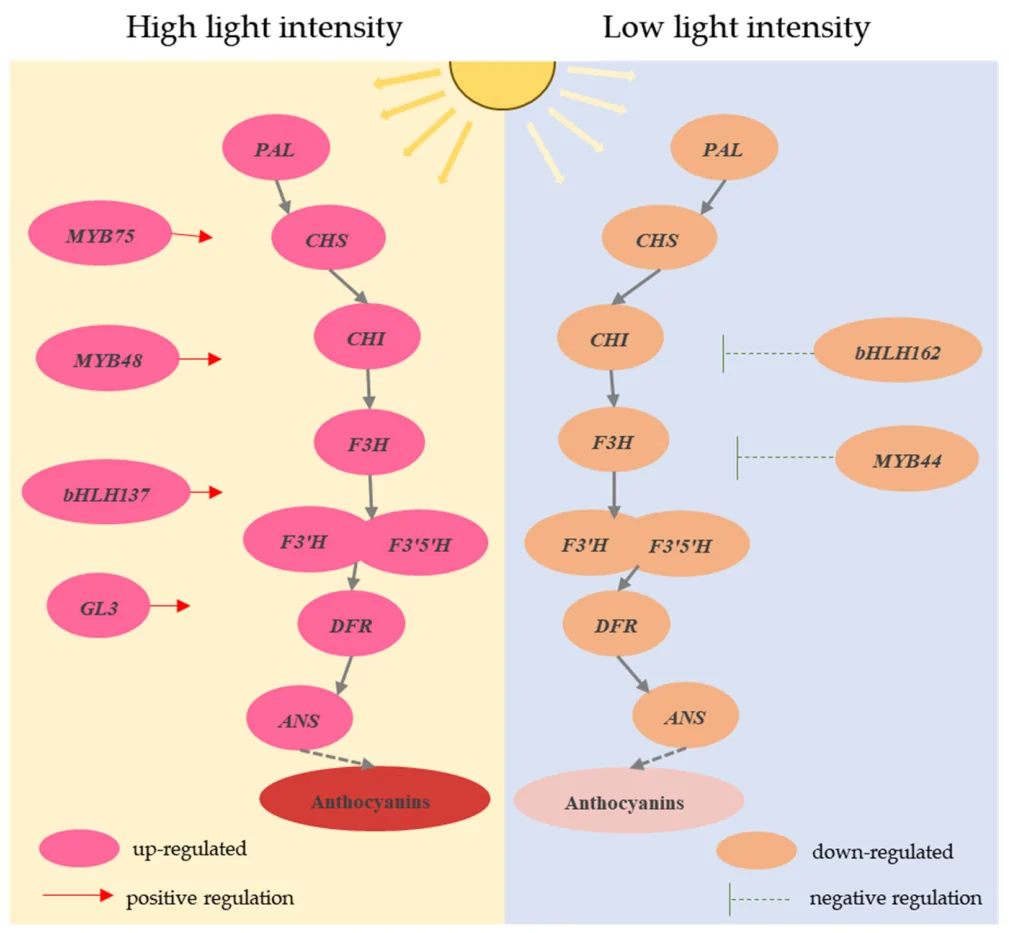
Potential mechanisms by which light intensity regulates anthocyanin biosynthesis in ‘Ziyan’.

Under varying light intensities, genes encoding enzymes involved in anthocyanin biosynthesis predominantly belonged to multi-gene families (Figure 5A). In the phenylpropanoid pathway, transcript levels of *PAL*, *C4H*, and *4CL* were elevated under high-light conditions. Notably, several key structural genes in the anthocyanin synthesis pathway—specifically *CHS* (TEA023340.1_gene), *F3’5’H* (TEA013315.1_gene), and *ANS* (TEA015762.1_gene)—exhibited lower expression under high-light-intensity compared to low-light-intensity treatment. In contrast, most other structural genes showed enhanced expression in response to high light intensity, including one *F3’H* gene (Camellia_sinensis_newGene_1267) and one *UFGT* gene (TEA026194.1_gene). This up-regulation of structural genes was consistent with the observed increase in corresponding enzyme activities under high-light-intensity conditions. In addition, two differentially expressed *arGST* genes (TEA025564.1 and Camellia_sinensis_newGene_45914) were identified. Analysis of gene expression related to competing flavonoid pathways, including *FLS*, *LAR*, and *ANR*, demonstrated a bifurcated response; approximately half of the gene family members exhibited high expression levels under low-light conditions, whereas the remaining members showed increased expression under high-light conditions. Notably, *FLS* (TEA010328.1_gene) was significantly up-regulated in response to high light intensity. Furthermore, the expression levels of *PAL*, *CHS2*, *F3H*, *F3’H*, *F3’5’H*, *UFGT*, and *FLS* increase with an increase in light intensity, while the expression level of *ANR* decreases (Figure 5B). These findings indicate that increasing the light intensity has a significant impact on the regulation of genes involved in anthocyanin biosynthesis. Furthermore, based on the transcription factors reported to regulate anthocyanin biosynthesis in other plant species, together with the transcription factors identified in this study (Figure 3), a potential mechanism by which light intensity regulates anthocyanin biosynthesis in ‘Ziyan’ was derived (Figure 6).

### 2.7. Anthocyanin Content in Different Treatments

**Figure 7:**
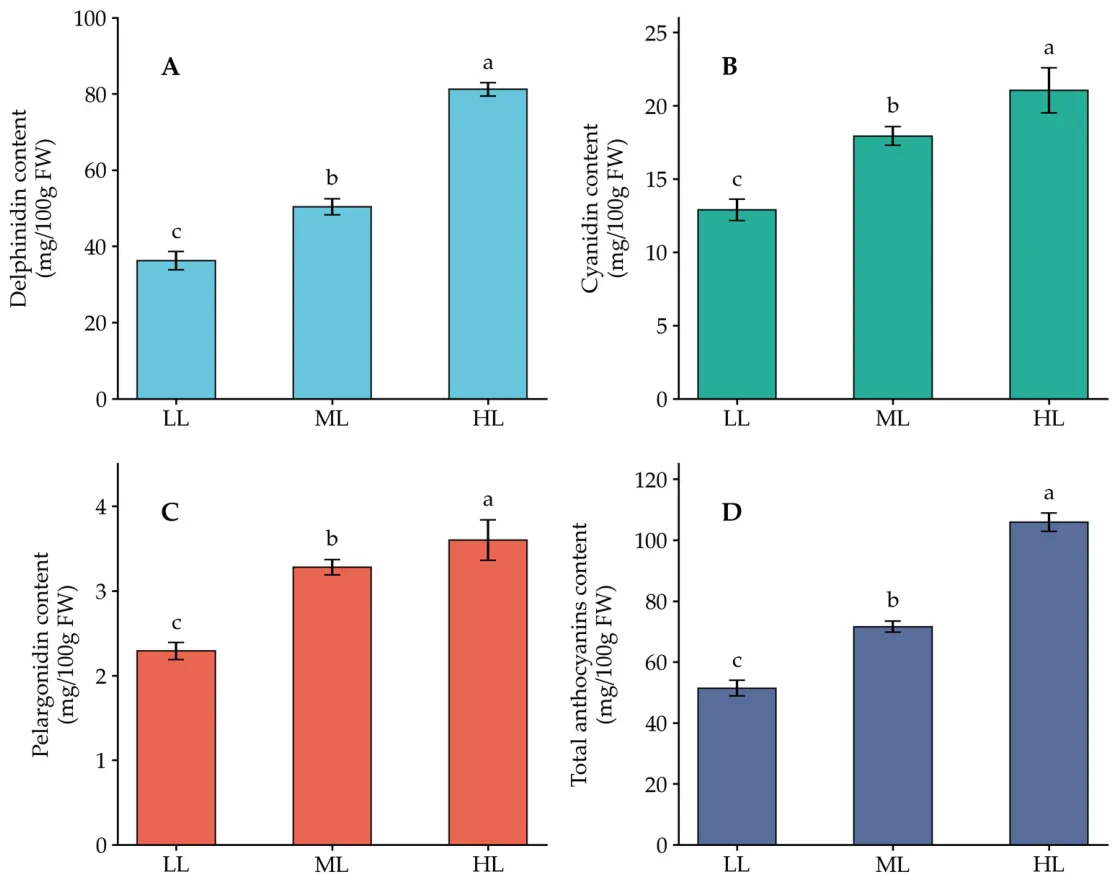
Anthocyanin contents under different light intensity treatments. (**A**) delphinidin cotent; (**B**) cyanidin cotent; (**C**) pelargonidin cotent; (**D**) total anthocyanins cotent. Different lowercase letters above the error bars indicate significant differences (*p* < 0.05).

The anthocyanin content in young shoots was significantly affected by different light intensity treatments (Figure 7). The delphinidin content ranged from 36.22 to 81.21 mg/100 g FW, the cyanidin content from 12.88 to 21.05 mg/100 g FW, the pelargonidin content from 2.29 to 3.60 mg/100 g FW, and the total anthocyanin content ranged from 51.39 to 105.85 mg/100 g FW. As light intensity increased, the contents of delphinidin, cyanidin, and pelargonidin increased significantly (*p* < 0.05). Specifically, under high-light-intensity conditions, the concentrations of delphinidin, cyanidin, pelargonidin, and total anthocyanins were 124.2%, 63.4%, 57.2%, and 105.9% higher, respectively, compared to those under low-light-intensity conditions. In the medium-light-intensity treatment, the contents of delphinidin, cyanidin, and pelargonidin were 39.2%, 39.2%, 43.2%, and 39.4% higher, respectively, than those observed under low-light-intensity conditions (Figure 7).

### 2.8. Catechin Contents in Young Shoots Under Different Light Intensities

**Figure 8:**
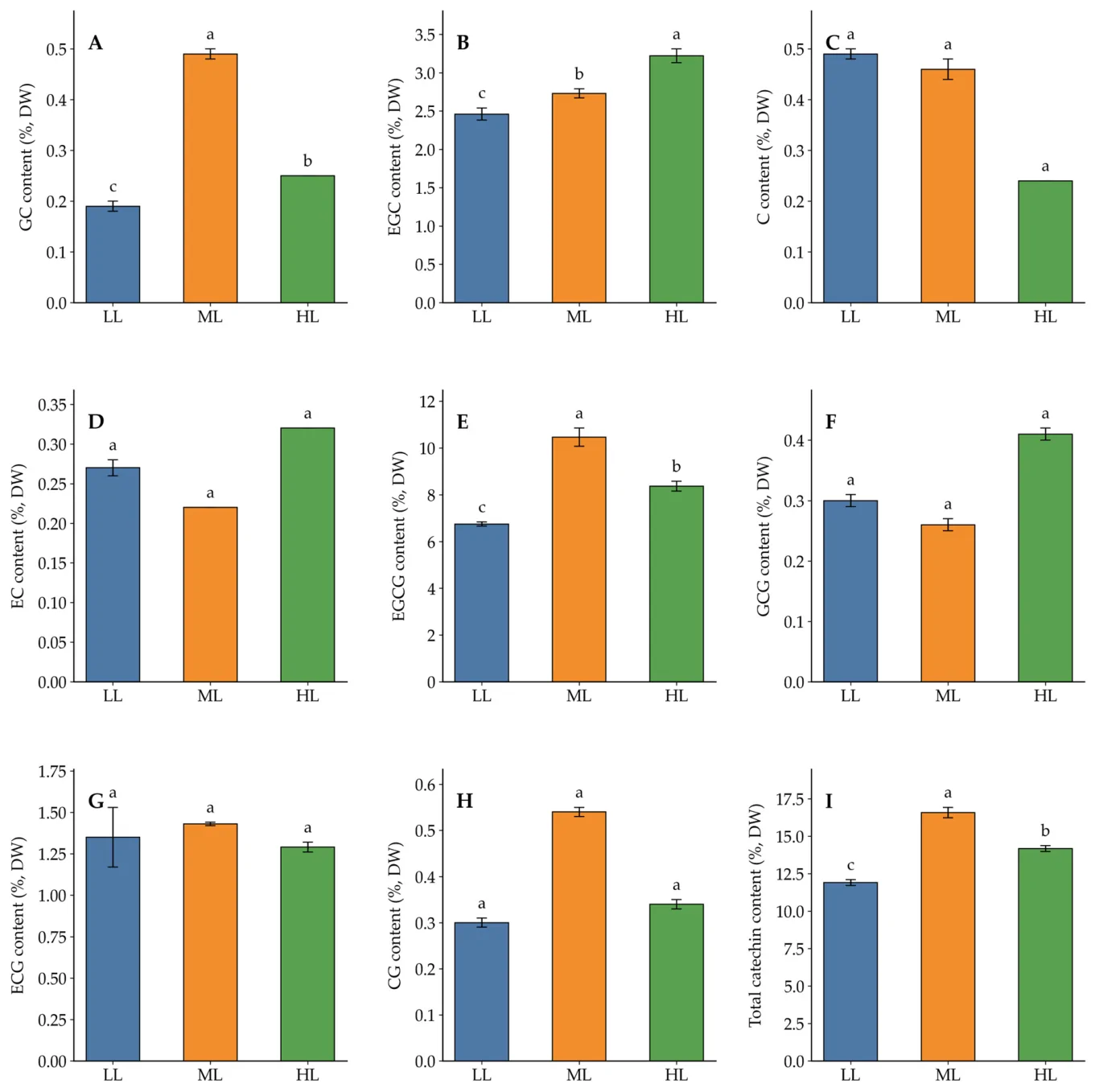
Effect of light intensity on catechin content in tea shoots. Panels (**A**–**I**) depict the catechin content under different light intensities: catechin (C), catechingallate (CG), epicatechin (EC), epicatechingallate (ECG), epigallocatechin (EGC), epigallocatechingallate (EGCG), gallocatechin (GC), and gallocatechingallate (GCG). Different lowercase letters following the numbers on the error bars indicate significant differences (*p* < 0.05).

The catechin content was analyzed in order to better understand the influence of light intensity on anthocyanin biosynthesis. Among the catechin constituents, EGCG exhibited the highest levels across all light intensity treatments, followed by EGC, while GCG levels were the lowest. The sequence of GC, EGCG, and total catechin content in shoots subjected to various treatments was ML > HL > LL, with differences that were statistically significant (*p* < 0.05) (Figure 8). Compared to low-light-intensity treatments, both medium- and high-light-intensity treatments significantly enhanced the contents of GC, EGC, EGCG, and CG (*p* < 0.05). Notably, under medium intensity, the aforementioned indicators increased by 157.9%, 11.0%, 55.0%, 80.0%, and 39.2%, respectively. Under high light intensity, the increases were 31.6%, 30.9%, 24.0%, 13.3%, and 19.1%, respectively. Overall, the individual catechins responded differentially to increasing light intensity: GC, EGC, EGCG, and CG increased under both ML and HL, whereas the responses of EC, ECG, and GCG were comparatively small or not statistically significant, and total catechin accumulation peaked under ML rather than increasing monotonically with light intensity. Thus, moderate light intensity appears optimal for catechin accumulation in ‘Ziyan’ shoots, whereas high light intensity favors anthocyanin accumulation, indicating divergent light intensity optima for the two major branches of flavonoid metabolism.

### 2.9. Activities of Main Enzymes in Anthocyanin Biosynthesis

Light intensity exerted a pronounced influence on the main enzyme activity in the anthocyanin biosynthesis pathway (Figure S5). As light intensity increased, the activities of enzymes such as CHS, CHI, F3H, F3’H, F3’5’H, DFR, and ANS in the anthocyanin biosynthesis pathway also increased. The activity of these enzymes under the high-light-intensity treatment was significantly increased compared to the control (*p* < 0.05), with increases of 35.25%, 35.15%, 12.99%, 35.93%, 26.43%, 46.24%, and 40.57%, respectively. The activities of CHS, F3’H, DFR, and ANS were also significantly different under different light intensities (*p* < 0.05). The anthocyanin reductase (ANR) activity decreased significantly with an increase in light intensity (*p* < 0.05). Compared to the control, ANR activity decreased by 23.56% and 7.07% under the high- and medium-light-intensity treatments, respectively (Figure S5II). The activity of leucoanthocyanidin reductase (LAR) was the highest under the medium-light-intensity treatment, significantly surpassing its activity as observed under the low and high light intensities by 91.65% and 76.73%, respectively, although no significant difference was noted between the low- and high-light-intensity treatments (*p* > 0.05).

Taken together, the concordant increases in the activities of the upstream anthocyanin biosynthetic enzymes (CHS, CHI, F3H, F3’H, F3’5’H, DFR, ANS), the significant reduction in ANR activity—the key enzyme diverting flux into the catechin branch—and the marked increase in anthocyanin content under HL provide mutually consistent, albeit correlative, evidence that high light intensity promotes a redistribution of flavonoid flux towards anthocyanin biosynthesis in the young shoots of ‘Ziyan’.

## 3. Discussion

Anthocyanins in plant tissues and organs play a crucial role in protecting the photosynthetic system and scavenging free radicals [25], and the biosynthesis and accumulation of anthocyanins in plants generally requires light. For instance, exposure to light can enhance the anthocyanin content in fruit peel [26,27]. Research has demonstrated that high light intensity enhances the expression of genes associated with the anthocyanin biosynthesis pathway, thereby increasing anthocyanin content [10,20]. Conversely, shade or low light intensity inhibits anthocyanin biosynthesis. The present study indicates that, as light intensity increases, both the total anthocyanin content and the component content in purple-tea shoots significantly increase, which aligns with existing findings [28,29]. Specifically, the total anthocyanin content in the new shoots of the ‘Ziyan’ variety subjected to high light intensity was 105. 88% higher than that under lower light intensity, suggesting that light intensity exerts a significant influence on anthocyanin content in purple-tea shoots. However, some studies have indicated that excessively high light intensity may reduce anthocyanin levels. The reason for the observed increase in anthocyanin content with higher light intensity in this study is that the maximum light intensity applied did not reach a threshold that would inhibit anthocyanin synthesis in tea plants [10,30,31]. Measurements of key enzyme activities involved in the anthocyanin biosynthesis pathway revealed that the activities of seven enzymes, namely CHS, CHI, F3H, F3’H, F3’5’H, DFR, and ANS, were significantly elevated under the high-light-intensity treatment (Figure S5). Under medium-light conditions, the activity of most of the enzymes involved in anthocyanin synthesis was higher than under low-light conditions. These findings suggest that increased light intensity enhances the activity of enzymes related to anthocyanin synthesis, thereby promoting anthocyanin production and resulting in a higher anthocyanin content in tea shoots.

From a physiological perspective, the high-light-induced anthocyanin accumulation observed in ‘Ziyan’ is consistent with the well-established photoprotective framework for anthocyanin function in vegetative tissues. When absorbed light energy exceeds the photosynthetic assimilation capacity, over-reduction in the photosynthetic electron transport chain increases the production of reactive oxygen species (ROS) at both photosystems. Vacuolar anthocyanins located in epidermal and sub-epidermal cells act as a molecular ‘sunscreen’ that absorbs the green and UV wavebands before they overexcite the chloroplasts [32,33], and in addition directly scavenge H_2_O_2_ and other ROS and chelate free metal ions, limiting Fenton-type hydroxyl-radical generation and thereby contributing to cellular redox homeostasis [33,34]. Anthocyanin accumulation under excess light is therefore considered one component of the integrated excess-light acclimation syndrome, operating alongside non-photochemical quenching and enzymatic antioxidant systems [35,36]. In this context, the coordinated up-regulation of the phenylpropanoid/flavonoid pathway, the increased activities of the biosynthetic enzymes, and the marked accumulation of anthocyanins under HL in ‘Ziyan’ can be interpreted as the executed acclimation program, whereas the ML-optimal accumulation of catechins is consistent with an allocation of carbon between photoprotective pigments and defense/storage metabolites. It should be noted that photosynthetic (e.g., Fv/Fm, NPQ) and oxidative-stress (e.g., H_2_O_2_ content) parameters were not measured in the present study; this physiological interpretation is therefore literature-based and correlational, and direct measurement of such parameters constitutes an important direction for future work.

Light quality [37,38] and photoperiod [39] have also been shown to significantly influence anthocyanin production in tea plants. This study focuses on the specific impact of light intensity on the anthocyanin biosynthetic pathway in purple-tea cultivar. A comparative transcriptomic analysis was conducted between shoots subjected to high and low light intensities. The results indicate that reduced light intensity significantly up-regulated the expression of most transcription factors associated with this pathway. Notably, Song et al. [24], through integrated metabolomic and transcriptomic analyses, systematically compared flavonoid metabolites and related gene expression differences between purple and green leaves of the purple-tea cultivar ‘Zijuan’ and revealed important regulatory mechanisms underlying anthocyanin biosynthesis. However, their study also suggested that the core transcription factors involved in anthocyanin regulation differ among tea cultivars. MYB44 plays a crucial role in the regulation of plant responses to abiotic and biotic stresses [40]. Previous studies have demonstrated that MYB44 acts as a negative regulator of anthocyanin biosynthesis [41,42]. Additionally, MYB73, MYB61, MYB14, MYB102, MYB48, and MYB75 have also been reported to be involved in regulating anthocyanin synthesis [43,44,45,46,47,48]. Notably, MYB75 has been confirmed to be involved in anthocyanin regulation in tea plants [49]. Within the bHLH family, bHLH137, bHLH162, bHLH35, bHLH51, bHLH92, and GL3 have been reported to be involved in the regulation of anthocyanin synthesis [50,51,52,53,54,55]. Although transcription factors of the WRKY gene family are predominantly associated with stress responses, WRKY11, WRKY23, WRKY40, and WRKY71 are involved in the regulation of anthocyanins [56,57,58,59]. Consequently, the aforementioned transcription factors exhibited differential expression in this study, suggesting they may be key regulators of anthocyanin synthesis in tea plants. From the perspective of structural genes involved in the anthocyanin synthesis pathway, it is clear that their expression levels are significantly higher under conditions of high light intensity compared to low light intensity. The analysis of catechin components reveals that catechin content reaches its maximum under medium light intensity, followed by high light intensity, with the lowest levels recorded under low-light conditions. These findings indicate that neither high nor low light intensities are conducive to catechin synthesis, which is consistent with the observed patterns of enzyme activity and gene expression profiles.

The TFBS motif enrichment analysis clarified the transcriptional regulatory landscape of light-induced anthocyanin biosynthesis. A single SPL/SBP module among up-regulated DEGs is notable because SPL transcription factors are well established as regulators of developmental phase transitions, organ morphogenesis, and secondary metabolism [22]. In *Arabidopsis thaliana*, SPL9 reportedly represses anthocyanin accumulation by destabilizing the MYB-bHLH-WD40 (MBW) activation complex, whereas miR156-mediated repression of SPL9 in juvenile leaves permits high anthocyanin accumulation [22]. The enrichment of SPL/SBP motifs in the promoters of light-induced genes in ‘Ziyan’ therefore initially appears inconsistent with this negative role, implying a context- or species-dependent function of SPL TFs in the light–anthocyanin axis. Recent functional evidence in tea supports such species specificity: the CsmiR156b–CsSPL9–CsMYB75 module fine-tunes anthocyanin biosynthesis by regulating CsANS1, in which CsmiR156b-targeted degradation of CsSPL9 weakens the CsSPL9–CsMYB75 protein interaction and thereby enhances MBW-complex-mediated activation of the CsANS1 promoter [23]. It should be emphasized that our motif enrichment result is a family-level prediction that cannot distinguish individual SPL members with activating versus repressing roles, nor their post-transcriptional regulation by miR156; the SPL/SBP module identified here therefore represents a strong candidate requiring functional validation in ‘Ziyan’ rather than a confirmed activator or repressor. Interestingly, the down-regulated DEGs were associated with multiple independent TF modules (NAC, HSF, EIL), which suggests that transcriptional repression under altered light intensity is mediated by diverse, parallel pathways rather than by a single repressor. The NAC family has been implicated in stress-responsive anthocyanin regulation, while HSF and EIL modules may reflect crosstalk among light, heat stress, and ethylene signaling. The detection of AHL13 as a broadly binding, non-specific TF underscores the importance of distinguishing genuine regulatory factors from background binders in motif enrichment analyses. Notably, the top-20 TFs from motif enrichment showed minimal overlap with the differentially expressed TFs identified in Section 2.5, which is expected because motif enrichment captures upstream regulators whose expression may not change, whereas DEG analysis identifies downstream effectors. It should be emphasized that these motif modules represent predictive, in silico associations between TF families and enriched promoter motifs; they do not demonstrate direct TF–target-gene interactions or an experimentally validated regulatory network, and the corresponding regulatory relationships remain to be tested experimentally (e.g., by EMSA, yeast one-hybrid, or transient activation assays).

The divergent light responses of anthocyanins and catechins also reveal a physiological trade-off in flavonoid carbon allocation that has practical implications for tea cultivation. Under HL, carbon and phenylpropanoid flux appear to be preferentially allocated to photoprotective anthocyanin accumulation, accompanied by reduced ANR activity; under ML, sufficient light supports high photosynthetic carbon gain while the photoprotective demand is lower, permitting greater flux into the catechin branch, consistent with the ML-optimal accumulation of GC, EGCG, and total catechins observed in this study. These results suggest that light intensity management can be used as a cultivation lever to direct tea quality attributes in ‘Ziyan’ and related purple-leaf cultivars: moderate shading or near-ML conditions favor catechin-rich green-tea quality, whereas higher light exposure or full sunlight favors anthocyanin-enriched purple-tea production. Defining cultivar-specific optimal light environments may therefore support site- and season-specific light management in purple-tea cultivation.

## 4. Materials and Methods

### 4.1. Plant Materials and Light Intensity Treatments

One-year-old, cutting-propagated purple-leaf tea plants of the ‘Ziyan’ cultivar (*Camellia sinensis*) were used in this study. Uniformly grown and healthy tea seedlings were selected and pruned to a uniform plant height of approximately 25 cm; after pruning, the plants were allowed to acclimate in the growth incubator for 5 days. The light intensity was set at three levels: low light intensity (100 μmol·m^−2^·s^−1^, LL), medium light intensity (200 μmol·m^−2^·s^−1^, ML), and high light intensity (400 μmol·m^−2^·s^−1^, HL). White light was provided by LED plant growth lamps (DUJIA-T5, 10 W, Guangdong Dujia Lighting Appliance Co., Ltd., Zhongshan, China). All the lamp tubes had identical power (10 W) and full spectrum, and were positioned approximately 10 cm above the top of the tea seedlings. All three light intensity treatments were conducted in separate plant growth chambers of the same model (Shanghai Sanfa Technology Co., Ltd., Shanghai, China), each chamber dedicated to one treatment. Other cultural conditions included a photoperiod of 14/10 h (light/dark), temperatures of 25/18 °C (light/dark), and a relative humidity of approximately 80%. The growth chambers and sampling methods used have been described in a previous study [30].

### 4.2. Color Measurement

The color of the second top leaf from the samples was quantified using a CM-2600d spectrophotometer (Konica Minolta, Tokyo, Japan) with a D65 illuminant and 45°/normal geometry. Each leaf was measured five times, avoiding the midrib. Color was recorded in the *L a b* color space, where *L* represents lightness, *a* indicates the green (−)-to-red (+) spectrum, and *b* represents the blue (−)-to-yellow (+) spectrum. The chroma (*C*) and hue angle (*h*°) were also calculated.

### 4.3. Anthocyanin and Catechin Content Measurement by HPLC

Fresh leaves were collected from ‘Ziyan’ tea plants for experimental purposes and promptly frozen in liquid nitrogen. The frozen leaf samples were then ground into a fine powder in liquid nitrogen. Approximately 0.2 g of this powder was immersed in 4 mL of methanol containing 1% (*v*/*v*) hydrochloric acid for 2 h at 4 °C. Following centrifugation at 5000 rpm for 10 min, the supernatant was transferred to a brown volumetric flask. The residues underwent two additional extractions using the same procedure, with 3 mL of extraction buffer and the soaking durations being 2 h and 12 h, respectively. After centrifugation at 5000 rpm for 10 min, all supernatants were combined into a brown volumetric flask. An acid hydrolysis–HPLC method was employed with slight modifications. Specifically, 0.2 mL of the extract was combined with 0.4 mL of 5 mol·L^−1^ HCl in a 1.5 mL tube. This tube was placed in a preheated dry bath at 90 °C for 30 min, and the hydrolyzed samples were immediately cooled in an ice bath. An Agilent 1260 HPLC system (Agilent, Palo Alto, CA, USA) and a Titank-C18 column (250 mm × 4.6 mm, 5 μm, Phenomenex Inc., Los Angeles, CA, USA) were utilized for the HPLC analysis. The eluents consisted of mobile phase A (water: acetonitrile: formic acid = 87:3:10, *v*/*v*/*v*) and mobile phase B (100% acetonitrile). The chromatographic conditions were as follows: 0 min, 15% B and 30 min, 30% B. The injection volume was 10 μL, the flow rate was 1 mL·min^−1^, and the C18 column was maintained at 35 °C. Anthocyanins were detected at 520 nm using an Agilent VWD detector. Five reference standards (peonidin chloride, pelargonidin chloride, delphinidin chloride, malvidin chloride, and cyanidin chloride) were procured from Chroma Dex (Los Angeles, CA, USA).

Catechins were extracted from one bud and two leaves in accordance with the Chinese National Standard GB/T8313-2018 [60]. High-performance liquid chromatography (HPLC) was conducted using an Agilent 1260 system (Agilent, Palo Alto, CA, USA) equipped with a Titank-C18 column (250 mm × 4.6 mm, 5 μm, Phenomenex Inc., Los Angeles, CA, USA). Mobile phase A comprised 9% acetonitrile and 2% acetic acid with 20 μg/mL EDTA, while mobile phase B consisted of 80% acetonitrile and 2% acetic acid with 20 μg/mL EDTA. The elution protocol was as follows: 100% A for 8 min, followed by a linear gradient to 68% A over 15 min, and subsequently maintained for 10 min. Reference standards for C, EC, GC, EGC, ECG, EGCG, GCG, and CG were procured from Sigma (Saint Louis, MO, USA). The HPLC conditions included a sample injection volume of 10 μL, a flow rate of 1 mL/min, a column temperature of 35 °C, and a detection wavelength of 278 nm. All HPLC quantifications of anthocyanins and catechins were performed on three independent biological replicates per treatment.

### 4.4. Measurement of Anthocyanin Biosynthesis-Related Enzyme Activity

The activities of the primary enzymes (CHS, CHI, F3H, F3′H, F3′5′H, DFR, LAR, ANS, and ANR) were assessed using plant-specific ELISA kits (Shanghai Fusheng Industry Co., Ltd., Shanghai, China). They are double-antibody sandwich ELISA kits produced against enzyme-specific antibodies, and the differences among the kits for the nine target enzymes reside only in the target-specific antibodies, while the assay principle is identical: the capture antibody pre-coated on the microplate binds the enzyme protein in the sample, a horseradish-peroxidase (HRP)-conjugated detection antibody binds a second epitope to form the antibody–antigen–HRP-antibody “sandwich” complex, and after addition of the TMB substrate the color developed (OD450) is proportional to the amount of enzyme protein in the sample. Quantification was performed against a standard curve calibrated by the manufacturer with purified standards. Accordingly, these assays quantify enzyme protein abundance (immunoreactive protein), reported as activity concentration in units of U/L, as shown on the y-axis of Figure S5.

A sample weighing 0.5 g was pulverized into a fine powder using liquid nitrogen, followed by the addition of 4.5 mL of phosphate-buffered saline (PBS, pH 7.4, 0.01 mol/L). The mixture was then centrifuged at 5000 rpm for 15 min at 4 °C. The supernatant was collected for enzyme activity assays and analyzed according to the manufacturer’s instructions. In brief, (a) all reagents were equilibrated to room temperature for 20 min prior to commencing the assay. (b) A volume of 10 μL of the test sample and 40 μL of sample diluent were added to the same microplate well. (c) Subsequently, 100 μL of horseradish peroxidase (HRP) conjugate reagent was introduced into each well. The microplate was immediately sealed with an adhesive strip and incubated for 60 min at 37 °C. (d) The liquid in the microplate wells was discarded, and the microplate was dried using absorbent paper. The wells were then washed with 400 μL of washing solution, allowed to stand for 1 min, and dried again on absorbent paper. This washing process was repeated five times. (e) A mixture of 50 μL of chromogen solution A and 50 μL of chromogen solution B was added to each well. The microplate was gently mixed and incubated immediately for 15 min at 37 °C in the dark. (f) Finally, 50 μL of stop solution was added to each well, and the optical density was measured within 15 min at 450 nm using a microplate reader.

### 4.5. RNA Extraction, Library Construction, and RNA-Seq

Total RNA was extracted from each sample following the protocol provided by the TRIzol Reagent (Invitrogen, Carlsbad, CA, USA). The RNA concentration was quantified using the NanoDrop 2000 spectrophotometer (Thermo Fisher Scientific, Waltham, MA, USA), and RNA integrity was evaluated with the RNA Nano 6000 Assay Kit on the Agilent Bioanalyzer 2100 system (Agilent Technologies, Palo Alto, CA, USA). RNA purity was assessed using the NanoPhotometer spectrophotometer (IMPLEN, Los Angeles, CA, USA). Three samples from each of the LL and HL treatments were used to construct six cDNA libraries, labeled as LL_1, LL_2, LL_3, HL_1, HL_2, and HL_3. For RNA sample preparations, 1.5 μg of RNA per sample was utilized as input material. Sequencing libraries were generated using the NEBNext UltraTM RNA Library Prep Kit for Illumina (NEB, Ipswich, MA, USA) in accordance with the manufacturer’s guidelines, with index codes added to assign sequences to each sample. Briefly, mRNA was isolated from total RNA using poly-T oligo-attached magnetic beads. Fragmentation was conducted using divalent cations at elevated temperatures in the NEBNext First Strand Synthesis Reaction Buffer (5X). First-strand cDNA synthesis was performed using random hexamer primers and M-MuLV Reverse Transcriptase, followed by second-strand cDNA synthesis using DNA Polymerase I and RNase H. Remaining overhangs were converted into blunt ends through exonuclease/polymerase activities. After adenylation of the 3′ ends of DNA fragments, the NEBNext Adaptor with a hairpin loop structure was ligated to prepare for hybridization. To select cDNA fragments 240 bp in length preferentially, the library fragments were purified using the AMPure XP system (Beckman Coulter, Beverly, MA, USA). Subsequently, 3 μL of USER Enzyme (NEB, USA) was applied to the size-selected, adaptor-ligated cDNA at 37 °C for 15 min, followed by 5 min at 95 °C before PCR. PCR was then conducted using Phusion High-Fidelity DNA polymerase, Universal PCR primers, and the Index (X) Primer. Finally, PCR products were purified using the AMPure XP system (Beckman Coulter, Brea, CA, USA), and library quality was assessed on the Agilent Bioanalyzer 2100 system. Clustering of the index-coded samples was performed on a cBot Cluster Generation System using the TruSeq PE Cluster Kit v4-cBot-HS (Illumina, San Diego, CA, USA) according to the manufacturer’s instructions. Following cluster generation, the library preparations were sequenced on an Illumina Hiseq 4000 platform, generating paired-end reads.

### 4.6. Transcriptome Analysis

Raw sequencing reads were filtered using a Perl script to remove low-quality reads, including those containing adaptors, those with more than 5% unknown nucleotides (N), and those with an average quality score below Q20. Clean reads were then aligned to the *Camellia sinensis* ‘Shuchazao’ reference genome with Tophat2 [61]. Following the removal of duplicate molecules from the alignment files, gene expression levels (FPKM) were calculated using Cufflinks(v2.2.1) [62].

### 4.7. Identification of Differentially Expressed Genes (DEGs)

Differential expression analysis between LL and HL was performed using DESeq2(v1.20.0) [63]. To address the possibility of multiple testing, statistical significance was assessed by controlling the false discovery rate (FDR). Genes with an absolute log_2_-fold change ≥ 1 and an FDR < 0.01 were considered significantly differentially expressed and were retained for subsequent analyses.

### 4.8. GO and KEGG Enrichment Analysis of DEGs

Gene Ontology (GO) enrichment analysis of the differentially expressed genes (DEGs) was conducted using the GOseq R package(v1.32.0) [64], which employs a Wallenius non-central hypergeometric distribution to correct for gene length bias. For pathway analysis, KEGG [65] enrichment was performed using KOBAS(v2.0) software [66].

### 4.9. Gene Expression Validation by qRT-PCR

The reliability of the RNA-seq data was verified by qRT-PCR using the CFX96 system (Irvine, CA, USA) with three replicates. Twelve genes involved in the flavonoid and chlorophyll biosynthetic pathways were selected for qRT-PCR validation. cDNA was synthesized from total RNA using a PrimeScript™ RT Kit with a gDNA Eraser (Takara, Kyoto, Japan), followed by qPCR amplification with SYBR Premix (Takara, Kyoto, Japan). The primer sequences used are provided in Table S5. *CsPTB* was used as the internal reference gene. Relative expression was calculated using the 2^−ΔΔCt^ method [67].

### 4.10. TFBS Motif Enrichment Analysis

TFBS motif enrichment in the promoters (1 kb upstream of the transcription start site) of DEGs was estimated using the ESDEG tools (https://github.com/ubercomrade/esdeg accessed on 2 August 2026) [68]. Motifs of known transcription factors were compiled from the JASPAR core database with plants (accessed on 2 August 2026), which have been shown to be suitable for cross-species TFBS analysis in plants. The analysis was performed separately for up-regulated and down-regulated DEG sets, with non-DEG promoters serving as the background. For pairwise motif redundancy assessment, the top-20 enriched motifs per direction were called against all 33,932 1 kb promoters using each motif’s ESDEG-selected ERR threshold, yielding binary target sets. Pairwise similarity between two motifs was assessed by Fisher’s exact test on the resulting 2 × 2 contingency table, and visualized as a heatmap of −log10(*p*), following the matrix-based framework of ESDEG.

## 5. Conclusions

This study shows that light intensity critically regulates anthocyanin biosynthesis in purple-leaf tea plants. Higher light intensity produced darker purple coloration, increased anthocyanin accumulation, and raised activities of key anthocyanin biosynthetic enzymes in the ‘Ziyan’ cultivar. Transcriptome analysis revealed that stronger light triggered widespread changes in gene expression and identified candidate transcription factors associated with light-responsive anthocyanin biosynthesis. TFBS motif enrichment indicated that up-regulated DEGs are predicted to be controlled by a unified SPL/SBP regulatory module, while down-regulated DEGs are associated with distinct TF modules (NAC, HSF, and EIL), suggesting complexity and asymmetry in the transcriptional network underlying light-responsive anthocyanin production. Together, these results propose a predictive regulatory model in which light intensity acts as a major driver of anthocyanin accumulation in ‘Ziyan’, providing an integrated framework for interpreting light-regulated pigmentation in purple-leaf tea, with practical implications for optimizing cultivation and candidate genes for improving anthocyanin-rich tea germplasm.

## Data Availability

The data presented in this study are included in the article/Supplementary Materials.

## Supplementary Materials

The following supporting information can be downloaded at https://www.mdpi.com/article/10.3390/plants15182805/s1. Table S1. Summary statistics of the sequencing data; Table S2. Genetic annotation database information; Table S3. TFBS motif enrichment among the top-20 enriched motifs in the 1-kb promoters of up-regulated DEGs; Table S4. TFBS motif enrichment among the top-20 enriched motifs in the 1-kb promoters of down-regulated DEGs; Table S5. Primers used for qRT-PCR analysis; Figure S1. Clustering heatmap of the samples; Figure S2. RT-qPCR validation of selected genes; Figure S3. Gene Ontology (GO) enrichment analysis in pairwise comparisons HL vs LL. Figure S4. KEGG enrichment analysis in pairwise comparisons of LL vs HL. Figure S5. Enzyme activity of key anthocyanin pathway under different light intensity treatments. Panels (A–I) depict the activities of anthocyanin biosynthesis-related enzymes. CHS, chalcone synthase; CHI, chalcone isomerase; F3H, flavone-3-hydroxylase; F3’H, flavonoid-3’-hydroxylase; F3’5’H, flavonoid-3’5’-hydroxylase; DFR, dihydroflavonol-4-reductase; ANS, anthocyanidin synthase; LAR, leucoanthocyanidin reductase; ANR, anthocyanidin reductase. Different lowercase letters on the error bars indicate significant differences (*p* < 0.05).
