# Supplementary material for: Integrated Transcriptomic and Biochemical Profiling Reveals the Regulatory Mechanism of Light Intensity-Induced Anthocyanin Biosynthesis in a Purple-Leaf Tea Cultivar
Source: Plants (Basel). 2026 Sep 13;15(18):2805. doi: 10.3390/plants15182805 (PMC13610245; doi:10.3390/plants15182805)
Supplement: Supplementary file 1 [file plants-15-02805-s001.zip › Supplementary .pdf]

Table S1 Summary statistics of the sequencing data

Sample name	Clean reads	Clean bases	GC Content	%≥Q30	Mapped Reads	Mapped Ratio
HL1	31,846,133	9,496,434,668	45.28%	92.61%	55,737,152	87.51%
HL2	35,429,631	10,550,256,880	45.23%	92.93%	61,755,011	87.15%
HL3	29,820,265	8,870,709,064	45.42%	92.92%	52,331,298	87.74%
LL1	32,468,382	9,689,375,428	44.91%	92.85%	56,724,611	87.35%
LL2	36,051,539	10,760,295,994	45.30%	92.50%	63,053,764	87.45%
LL3	30,596,516	9,120,013,200	45.40%	92.66%	53,567,133	87.54%

HL1, HL2, and HL3; LL1, LL2, and LL3 represent three biological replicates of high light intensity and low light treatment, respectively.

Table S2 Genetic annotation database information

Database	Description	Homepage
NR	non-redundant protein sequence database	ftp://ftp.ncbi.nih.gov/blast/db/
Swiss-Prot	A manually annotated, non-redundant protein sequence database	http://www.uniprot.org/
KEGG	The database of Kyoto Encyclopedia of Genes and Genomes	http://www.genome.jp/kegg/
GO	Gene Ontology database	http://www.geneontology.org/
Pfam	The database of Homologous protein family	http://pfam.xfam.org/
eggNOG	evolutionary genealogy of genes: Non-supervised Orthologous Groups database	https://eggnogdb.org/
COG/KOG	The database of Clusters of Orthologous Groups of proteins	http://www.ncbi.nlm.nih.gov/COG/
	The database of Clusters of Protein homology	http://www.ncbi.nlm.nih.gov/KOG/

Table S3 TFBS motif enrichment among the top-20 enriched motifs in the 1-kb promoters of up-regulated DEGs

Rank	−lg(padj)	TF Class	TF Family	TF Name	JASPAR ID
1	297.19	C3H(C),C2HC zinc-fingers like factors	SBP	SPL3B	MA2442.1
2	297.19	C3H(C),C2HC zinc-fingers like factors	SBP	SPL3D	MA2443.1
3	297.19	C3H(C),C2HC zinc-fingers like factors	SBP	SPL5A	MA2446.1
4	297.19	C3H(C),C2HC zinc-fingers like factors	SBP	SPL7B	MA2451.1
5	297.19	C3H(C),C2HC zinc-fingers like factors	SBP	SPL9D	MA2455.1
6	297.19	C3H(C),C2HC zinc-fingers like factors	SBP	SPL5D	MA2448.1
7	297.19	C3H(C),C2HC zinc-fingers like factors	SBP	SPL4A	MA2444.1
8	297.19	C3H(C),C2HC zinc-fingers like factors	SBP	SPL2A	MA2439.1
9	297.19	C3H(C),C2HC zinc-fingers like factors	SBP	SPL16A	MA2434.1
10	297.19	C3H(C),C2HC zinc-fingers like factors	SBP	SPL16D	MA2435.1
11	297.19	C3H(C),C2HC zinc-fingers like factors	SBP	SPL15A	MA2431.1
12	297.19	C3H(C),C2HC zinc-fingers like factors	SBP	SPL14B	MA2430.1
13	297.19	C3H(C),C2HC zinc-fingers like factors	SBP	SPL13B	MA2428.1
14	297.19	C3H(C),C2HC zinc-fingers like factors	SBP	SPL13D	MA2429.1
15	297.19	C3H(C),C2HC zinc-fingers like factors	SBP	SPL11B	MA2426.1
16	297.19	C3H(C),C2HC zinc-fingers like factors	SBP	SPL12B	MA2427.1
17	297.19	C3H(C),C2HC zinc-fingers like factors	SBP	SPL2B	MA2440.1
18	297.19	C3H(C),C2HC zinc-fingers like factors	SBP	SPL1D	MA2438.1
19	297.19	C3H(C),C2HC zinc-fingers like factors	SBP	SPL18A	MA2436.1
20	297.19	C3H(C),C2HC zinc-fingers like factors	SBP	SPL19B	MA2437.1

Table S4 TFBS motif enrichment among the top-20 enriched motifs in the 1-kb promoters of down-regulated DEGs.

Rank	−lg(padj)	TF Class	TF Family	TF Name	JASPAR ID
1	11.5013	C3H(C),C2HC zinc-fingers like factors	SBP	SPL7A	MA2450.1
2	8.36297	EIL	EIL	EIN3	MA2634.1
3	8.20695	GCM domain factors	NAC	NAC031	MA2045.2
4	6.48512	C3H(C),C2HC zinc-fingers like factors	SBP	SPL1D	MA2438.1
5	6.01593	GCM domain factors	NAC	NAC038	MA2033.2
6	6.01593	GCM domain factors	NAC	NAC075	MA2035.2
7	6.01593	Heat shock factors	HSF	HSFA4A	MA1759.2
8	3.2224	AP2/EREBP	ERF/DREB	Solyc03g124110	MA2715.1
9	2.60273	GCM domain factors	NAC	NAC010	MA2009.2
10	2.05569	GCM domain factors	NAC	NAC046	MA0936.1
11	2.05569	Heat shock factors	HSF	HSFA6A	MA1760.1
12	1.89625	GCM domain factors	NAC	NAC058	MA0938.3
13	1.8947	GCM domain factors	NAC	NAC079	MA0939.1
14	1.8947	A.T hook factors	AHL	AHL13	MA2374.1
15	1.8947	GCM domain factors	NAC	NAC020	MA2006.2
16	1.77708	EIL	-	EIL3	MA1751.2
17	1.77708	C3H(C),C2HC zinc-fingers like factors	SBP	TraesCS7B02G142200	MA2750.1
18	1.7299	C3H(C),C2HC zinc-fingers like factors	SBP	SPL18A	MA2436.1
19	1.7299	EIL	-	TEIL	MA2362.1
20	1.70556	EIL	-	eil1	MA2712.1

"-" indicates not annotated in the JASPAR database.

Table S5 Primers used for qRT-PCR analysis

Gene id	Gene name	Sequence （5'→3'）	
TEA014056.1_gene	*PAL*	Forward primer	CAAAACAAGACCGGTACGCC
		Reverse primer	TCGATTAGCGGGTTGTCGTT
TEA034042.1_gene	*CHS*	Forward primer	GCTCGGGTCCTAGTTGTGTG
		Reverse primer	AACCTCGGGAATTGGGTCTG
Camellia_sinensis_newGene_1644	*F3H*	Forward primer	AGCGGTGACGGAGAAGTACA
		Reverse primer	CCAATCTCATCGCCTCCGAC
Camellia_sinensis_newGene_1267	*F3′H*	Forward primer	GGGGACCGATGAGGTTTAGG
		Reverse primer	ATCACCAAGGCACCTCTCCA
TEA026296.1_gene	*F3′5′H*	Forward primer	ACCCATCAGTTCCCCTCAGT
		Reverse primer	TCTCCCACACATCAGGGTCT
TEA032730.1_gene	*DFR*	Forward primer	CGCGGCTATATTGTTCGTGC
		Reverse primer	ATGGCCTCATCAAAGCTCCC
TEA015769.1_gene	*ANS*	Forward primer	GGGCCACAAGTGCCTACAAT
		Reverse primer	TCCCGACACCTCTCTCTCAC
TEA022960.1_gene	*ANR*	Forward primer	GAAGGCCGCATGTGTTGTTG
		Reverse primer	GTTCATCGGTGAGGTCTGCT
TEA025792.1_gene	*UFGT*	Forward primer	GCGGTGGTTTCTTACGTGTT
		Reverse primer	GGGAAGCCAATTGGTGGTTG
TEA010328.1_gene	*FLS*	Forward primer	GGAGTTGGAGAGGGTGCAAG
		Reverse primer	GTCGTCCGAGTCATTGCTGA
TEA026458.1_gene	*LAR*	Forward primer	GTGTCAAGCCCTTGTGTTGG
		Reverse primer	GCTTGAGCAATGAACTCGCC
TEA026253.1_gene	*HEMA*	Forward primer	CTCGTACCAGACGAGCCCTA
		Reverse primer	CTTGAGTTGTTCGAGCGCAG
TEA012308.1_gene	*DVR*	Forward primer	GTCATGCCTTGCTAGCCGAA
		Reverse primer	ATGTGAAGCCCCACGATTCC
Camellia_sinensis_newGene_37489	*CAO*	Forward primer	CCTTTGCCAAGGGATGGAGT
		Reverse primer	GTATCCTTGGAGGCCGGATG
TEA021772.1_gene	*NYC*	Forward primer	GGCATGGTTCTGACGGACTT
		Reverse primer	CCCGCATCCGTGGAACTAAA
TEA002246.1_gene	*PTB*	Forward primer	ATGCCGGCTCAATCTTCTGT
		Reverse primer	CCTGATTGCACAAGGGGTGA

PTB as the reference gene

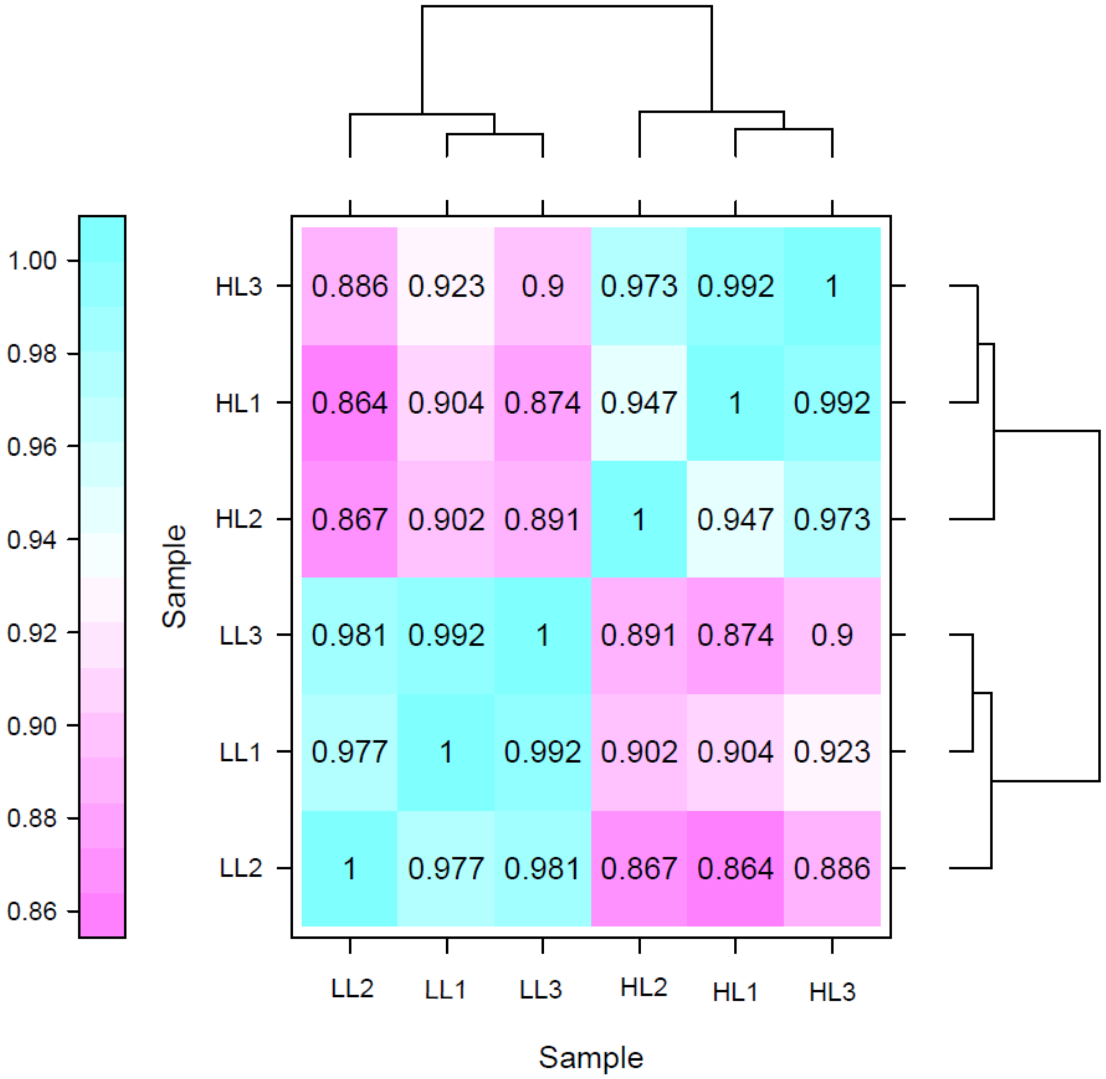

Figure S1. Clustering heatmap of the samples.

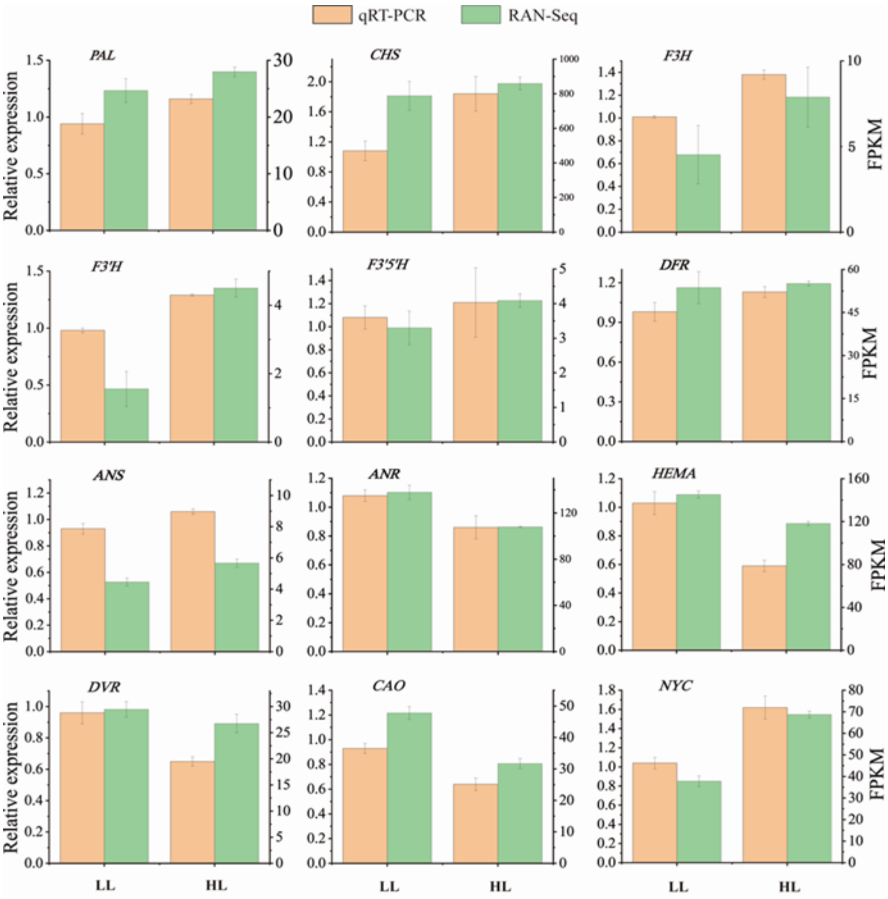

Figure S2. RT-qPCR validation of selected genes

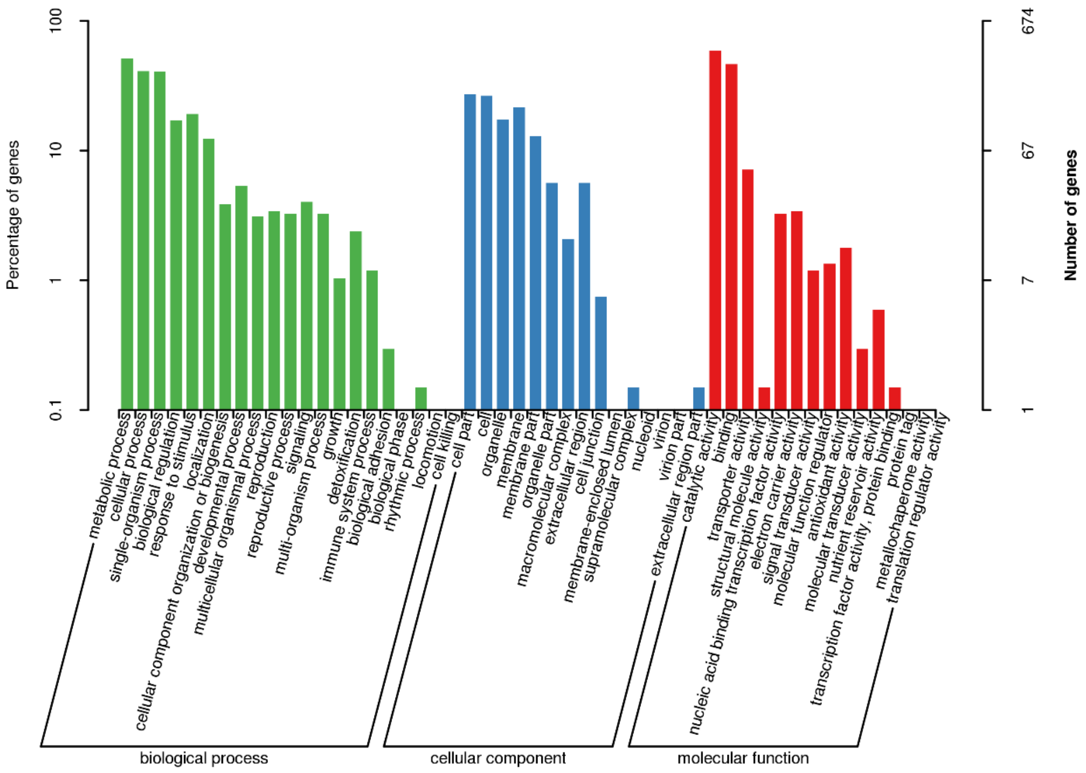

Figure S3. Gene Ontology （GO） enrichment analysis in pairwise comparisons HL vs LL.

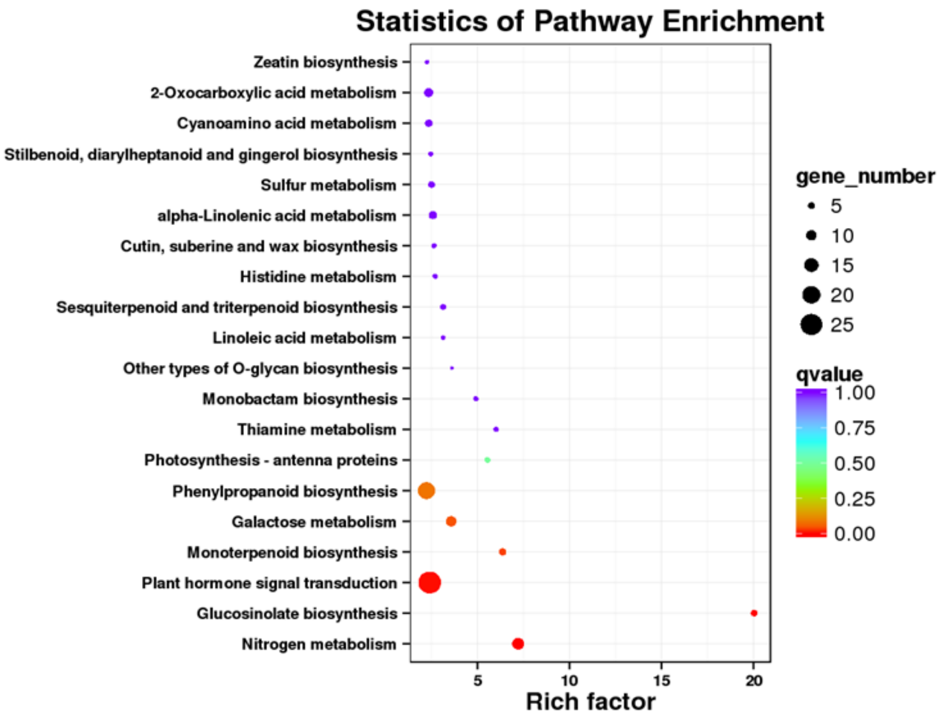

Figure S4. KEGG enrichment analysis in pairwise comparisons of LL vs HL.

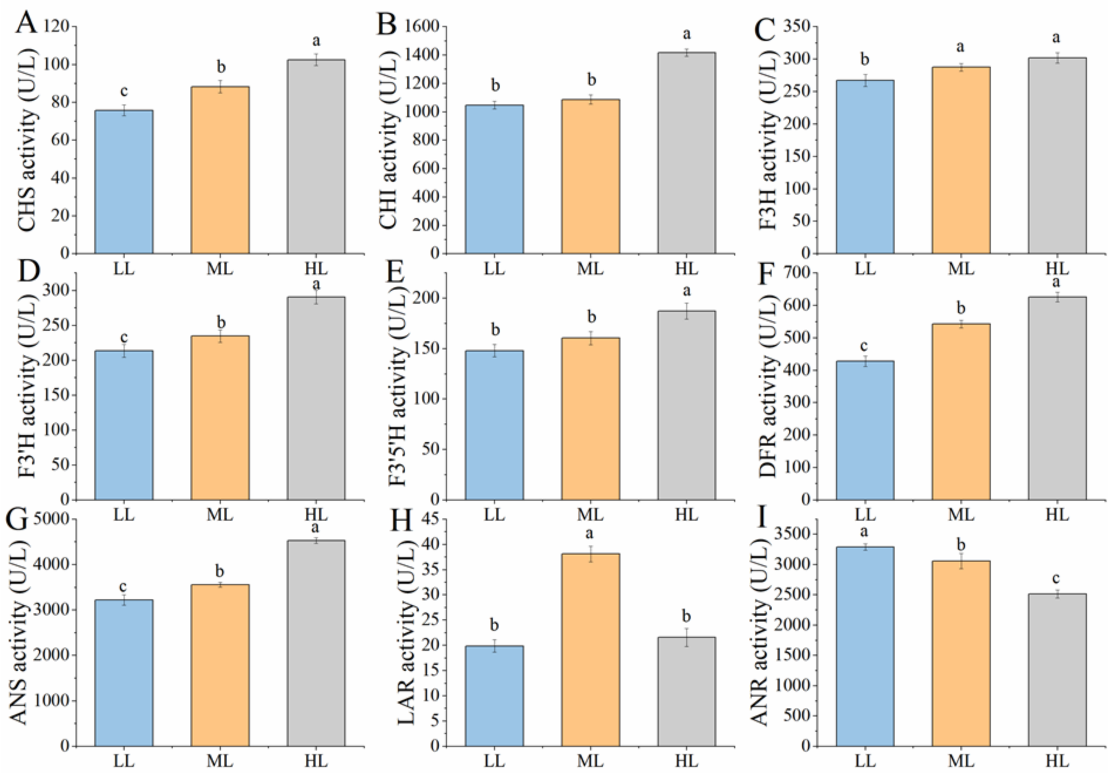

Figure S5. Enzyme activity of key anthocyanin pathway under different light intensity treatments. Panels (A–I) depict the activities of anthocyanin biosynthesis-related enzymes. CHS, chalcone synthase; CHI, chalcone isomerase; F3H, flavone-3-hydroxylase; F3'H, flavonoid-3'-hydroxylase; F3'5'H, flavonoid-3'5'-hydroxylase; DFR, dihydroflavonol-4-

reductase; ANS, anthocyanidin synthase; LAR, leucoanthocyanidin reductase; ANR, anthocyanidin reductase. Different lowercase letters on the error bars indicate significant differences ($p < 0.05$).
